# Structural evolution of lotus seed resistant starch during *in vitro* fecal fermentation in food-allergic rats modulates gut microbiota and SCFAs

**DOI:** 10.3389/fnut.2026.1870906

**Published:** 2026-06-30

**Authors:** Lanxin Li, Xiangfu Jiang, Yingyu Zhao, Baodong Zheng, Bee K. Tan, Hongliang Zeng, Yi Zhang

**Affiliations:** 1 College of Food Science, Fujian Agriculture and Forestry University, Fuzhou, China; 2 Fujian Provincial Key Laboratory of Quality Science and Processing Technology in Special Starch, Fujian Agriculture and Forestry University, Fuzhou, China; 3 Key Laboratory of Subtropical Characteristic Fruits, Vegetables and Edible Fungi Processing (Co-construction by Ministry and Province), Ministry of Agriculture and Rural Affairs, Fuzhou, China; 4 Department of Cardiovascular Sciences and Diabetes Research Centre, University of Leicester, Leicester, United Kingdom

**Keywords:** lotus seed resistant starch, gut microbiota, SCFAs, structural characteristics, Bifidobacterium

## Abstract

This study investigated the effects of lotus seed resistant starch type 3 (LRS3) on the gut microbiota and metabolism of normal and food-allergic rats, as well as the structural evolution of LRS3 during fermentation, using an *in vitro* simulated fermentation model. Results revealed a distinct temporal pattern in microbial degradation of LRS3. Microorganisms preferentially degraded the amorphous regions, leading to the preferential consumption of the outermost short chains (A-chains) of amylopectin and a significant increase in the amylose content to 51.11%. As fermentation progressed, microbial activity extended to the crystalline regions, causing their breakdown and a decrease in relative crystallinity to 37.8%. These structural changes coincided with marked shifts in the gut microbiota, characterized by selective enrichment of Bifidobacterium and reduction of *Escherichia coli*-Shigella species. Correlation analysis revealed a significant positive correlation between Bifidobacterium abundance and acetate. LRS3 alleviated allergic reactions by modulating gut microbiota through its structural decomposition, promoting beneficial bacteria and acetate production. This study provided a mechanistic foundation for developing functional foods targeting the microbiota.

## Graphical Abstract

**Figure:**
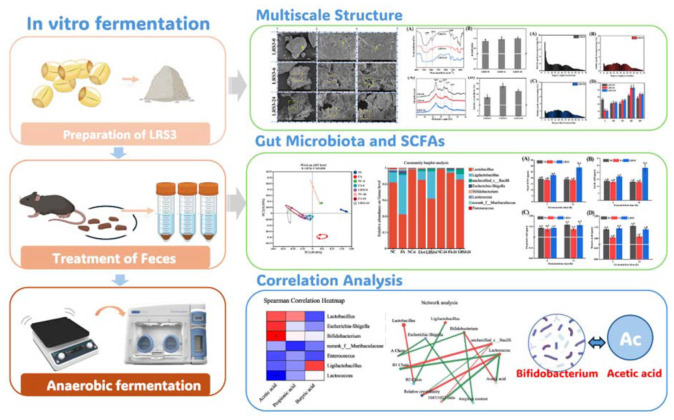
Structural evolution of lotus seed resistant starch during in vitro fecal fermentation in food-allergic rats modulates gut microbiota and SCFAs.

## 1 Introduction

Lotus seeds are perennial aquatic herbaceous plants belonging to the Nymphaeaceae family. They are high-starch foods, with starch content exceeding 50% (based on dry matter content of lotus seeds). The proportion of amylose in the starch reaches as high as 40%, making it a distinctive starch with relatively high amylose content that readily forms resistant starch (RS) (1). The tolerance of RS to food processing conditions and its superior functional properties compared to other non-starch polysaccharides have become a topic of interest in food science research. As a novel dietary fiber, RS is fermented and decomposed by microbial flora as a carbon source, thereby regulating the composition of gut microbiota and producing metabolites such as short-chain fatty acids (SCFAs) (2). It extensively participates in various physiological activities within the body and exerts its biological effects. Our previously studied lotus seed resistant starch (LRS3) was a type 3 resistant starch prepared through gelatinization-retrogradation (3). Research has shown that LRS has significant advantages in maintaining gut ecology and regulating glucose and lipid metabolism due to its resistance to digestion and its probiotic properties (4).

In recent years, food allergy (FA) has become a prevalent clinical condition, drawing widespread attention and concern from the food science and nutrition communities (5). This is due to multiple factors, such as food additives, food variety, and changes in dietary patterns. FA is an abnormal immune response triggered by the ingestion of certain foods or food components (6). Mild cases may cause skin hives or gastrointestinal and respiratory spasms, while severe reactions can lead to asthma attacks, anaphylactic shock, life-threatening conditions, or even death (7). However, current treatment methods involve avoiding exposure to allergenic foods and administering emergency medications, with no superior therapeutic options available. Common anti-allergy drugs include antihistamines and corticosteroids, but these may cause side effects such as fever, fatigue, and rashes (8). Population-based studies have demonstrated a close relationship between dietary fiber intake and susceptibility to FA, with low-fiber diets significantly increasing the incidence of allergic diseases (9). Research has shown that a high-fiber diet can protect mice from peanut allergies by influencing their gut microbiota and the production of short-chain fatty acids (10).

The gut microbiota constitutes a vital microbial community within the human body, participating in the development and regulation of the immune system, as well as the absorption and metabolism of nutrients (11). Dietary fiber suppresses FA by regulating gut microbiota and producing metabolites such as SCFAs through microbial metabolism. The most potent suppression occurs when both pathways are activated, the absence of either pathway weakens the inhibitory effect (10). However, the interaction mechanisms among LRS3, FA, gut microbiota, and their metabolic products-SCFAs-remain poorly understood. *In vitro* fermentation models serve as valuable tools for assessing the metabolic capabilities of human gut microbiota under anaerobic conditions (12). By providing a controlled environment, these models help to elucidate the specific interactions between polysaccharides and microorganisms. This guides the development of functional foods based on microbial metabolism (13). Based on this, this study employs an *in vitro* fecal microbiota fermentation model to systematically analyze the structural degradation patterns of LRS3 and explore its structure-activity relationship with SCFAs production. This approach aims to reveal the potential mechanism by which this starch modulates SCFA dynamics by regulating the composition of the gut microbiota, thereby affecting FA. This study aims to precisely identify functional food factors that improve susceptibility to FA, providing theoretical support for the development of microbe-directed foods.

## 2 Materials and methods

### 2.1 Materials and reagents

The lotus seeds were obtained from Fujian Green Field Co., Ltd. (Fujian, China). Raw materials for basic fermentation media were provided by Shanghai Macklin Biochemical Co., Ltd. (Shanghai, China) and included tryptone, yeast extract, hemin, and resazurin. Glucose (GLU) was obtained from Sinopharm Group Chemical Reagent Co., Ltd. (Shanghai, China). Acetic acid, propionic acid, and butyric acid reference standards were purchased from Sigma-Aldrich Co. All other chemical reagents used in this study were of analytical grade. These chemicals were used directly without further purification.

### 2.2 Preparation of LRS3

For LRS3 preparation and purification, see our previous research (14). The starch content of LRS3 in this study was > 95 %, its resistant starch content was 57.6%, moisture content was < 2 %, lipid content was 0.1 –0.2%, and protein content was approximately 3–6% (15). Briefly, lotus seeds were pulverized in a blender with an appropriate amount of distilled water. The mixture was allowed to settle, and the precipitate was collected. The precipitate underwent water washing and alcohol washing to obtain lotus seed starch. A 30% lotus seed starch suspension was prepared at a 3:7 ratio. The suspension was gelatinized in an autoclave and then cooled at 4°C for 24 h to allow for retrogradation. It was then purified using α-amylase and glucose amylase. Finally, purified LRS3 was obtained through centrifugation, washing, and drying.

### 2.3 *In vitro* fecal fermentation

Three-week-old Brown Norway (BN) rats (Specific pathogen-free, SPF grade) were purchased from Beijing Weitong Lihua Laboratory Animal Technology Co., Ltd. (license no. SCXK (Beijing) 2021–0006). Animals were housed in polycarbonate cages (SHINVA, Shandong Xinhua Medical Instrument Co., Ltd.; dimensions: 475 mm × 350 mm × 200 mm) containing autoclaved corncob bedding (Fuzhou Wushi Animal Testing Co., Ltd.). Each cage contained three rats (*n* = 6 per group, two cages per group). Environmental conditions were maintained at 22–24°C, 30–60% relative humidity, and a 12h light/dark cycle. Rats had *ad libitum* access to standard irradiated rodent chow (Fuzhou Wushi Animal Testing Co., Ltd.) and autoclaved tap water provided in polycarbonate bottles with stainless steel sipper tubes (replaced every 48h). BN rats (FA group) were sensitized by continuous gavage of 1 mL of 1 mg/mL ovalbumin (OVA) for 41 days, followed by a high-dose (1 mL of 100 mg/mL) gavage on day 42. Normal rats (NC group) received gavage of an equivalent volume of physiological saline. On day 42 of the experiment, following high-dose OVA stimulation, fecal samples were collected from FA rats (FA group) and normal rats (NC group). Animal experiments were approved by the Ethics Committee of Fujian Agriculture and Forestry University, approval number PICASFAFU25131. Rats were euthanized by intraperitoneal injection of an overdose of 2% sodium pentobarbital (150mg/kg). After injection, while the animal was still deeply anesthetized but before cardiac arrest, blood was collected via retro-orbital sinus puncture. Death was confirmed by the absence of respiration, heartbeat, and pupillary light reflex.

Following a modified previous protocol, rat fecal microbiota inoculum was prepared and *in vitro* fermentation was conducted (16). The entire procedure is performed under anaerobic conditions. The rat fecal inoculum was prepared by homogenizing fresh feces of same group in 0.1 M phosphate buffer (pH 7.2–7.4), followed by sequential centrifugation: low-speed centrifugation (200 × g, 5 min) to remove solids, and high-speed centrifugation (9,000 × g, 5 min) to harvest microbial cells. The pellet was washed and resuspended in sterile PBS to form the primary fecal suspension. This suspension was then activated by inoculating at 16% (v/v) into a pre-culture medium containing 3 g/L tryptone, 1.5 g/L yeast extract, 3 g/L NaCl, 1.8 g/L maltose, and 1.5 g/L glucose, and incubating anaerobically for 18 h to generate the primal bacterial (PB) culture. For the main fermentation, the PB culture was inoculated at 16% (v/v) into a sterile basal medium composed of 0.5 g/L bile salt, 4 g/L tryptone, 2 g/L yeast extract, 0.1 g/L NaCl, 2 g/L NaHCO_3_, 0.01 g/L MgSO_4_⋅7H_2_O, 0.01 g/L CaCl_2_⋅6H_2_O, 0.04 g/L K_2_HPO_4_, 0.04 g/L KH_2_PO_4_, 0.5 g/L L-cysteine, 2 mL/L Tween-80, 50 mg/L hemin, and 4 mL/L of 0.025% resazurin solution. This basal medium was supplemented with 2% (w/v) of the respective carbon sources (LRS3 group: LRS3, FA group: GLU, NC group: GLU). The fermentation was carried out anaerobically at 37°C, with samples collected at 0, 6, 24 h for analysis. Based on fermentation time (0, 6, and 24 h), samples were designated as follows: LRS3 group: LRS3-0, LRS3-6, and LRS3-24; FA group: FA, FA-6, and FA-24; NC group: NC, NC-6, and NC-24.

### 2.4 Determination of sugar derivatives

The composition ratio of glucose to maltose 2–7 in the sample was determined by ion chromatography. Analysis was performed on a ThermoFisher ICS5000 system equipped with a Dionex CarboPac™ PA200 column (3 × 150 mm). Samples were diluted 50-fold (20 μL sample + 980 μL water), centrifuged (12,000 rpm, 5 min), and filtered through a 0.22 μm membrane prior to injection. Chromatographic conditions were as follows: mobile phase consisted of water, 500 mM NaOH, and 65 mM NaOH/1025 mM sodium acetate solution; flow rate was 0.3 mL/min; injection volume was 25 μL; column temperature was 30°C. All standards were prepared at specified concentrations (17).

### 2.5 Scanning electron microscopy observation

For scanning electron microscopy (SEM) analysis, the dried starch samples were fixed onto aluminum stubs with conductive adhesive. Subsequently, the samples were gold-sputtered. Microstructural observations were performed with a scanning electron microscope (Gemini SEM560, MERLIN Compact, Germany) at 1.5 kV and 1,000x, 5,000x, 10,000x magnification.

### 2.6 Determination of amylose content

Amylose content was determined by a modified iodine colorimetry method (18). A standard curve was generated from amylose-amylopectin blends containing 0, 9.7, 16.1, and 25.5% amylose. For sample analysis, 10 mg of starch was weighed and dissolved in 100 μL of ethanol and 900 μL of NaOH, followed by heating in a boiling water bath for 13 min for gelatinization. After cooling and diluting to 10 mL, the solution stood for 10 min. Subsequently, 0.5 mL of the supernatant was taken for the chromogenic reaction by adding 0.1 mL acetic acid and 0.2 mL KI solution, then diluting to 10 mL. Following a 10-min incubation in the dark, the absorbance was recorded at 620 nm on a Multiskan GO spectrophotometer (Thermo Fisher Scientific, United States).

### 2.7 Chain length distribution of amylopectin

Amylopectin chain length distribution was analyzed by ion chromatography (ICS5000 + , Thermo Fisher Scientific, United States) coupled with an electrochemical detector. Starch samples (10 mg) were gelatinized in 5 mL of water via a 60-min treatment in a boiling bath with intermittent agitation. The gelatinized starch was then subjected to enzymatic debranching by incubation with isoamylase (10 μL, 1,400 U) in a sodium acetate buffer (50 μL, 0.6 M, pH 4.4) containing sodium azide (10 μL, 2% w/v) at 37 °C for 24 h. The released chains were subsequently reduced by adding sodium borohydride to 0.5% (w/v) and standing for 20 h. Prior to injection, the dried (under nitrogen) sample was reconstituted in 30 μL of 1 M NaOH, diluted with 570 μL of water, and centrifuged (12,000 rpm, 5 min) to collect the supernatant.

### 2.8 X-ray diffraction

The crystalline structure of the samples was investigated by X-ray diffraction (XRD) using a SmartLab diffractometer (Rigaku Corporation, Japan). The XRD patterns were acquired by scanning the diffraction angle (2θ) from 5° to 35° at a scan rate of 2°/min. The measurement was performed under operational conditions of 40 kV and 40 mA. Crystallinity (%) was calculated using Equation (1).


$$\mathrm{Crystallinity}\left(\%\right)=\frac{\mathrm{Ac}}{\mathrm{Aa}+\mathrm{Ac}}$$ (1)

where Ac represents the crystalline area and Aa denotes the amorphous area on the X-ray diffractograms.

### 2.9 Fourier transform infrared spectroscopy

FT-IR analysis was performed using a Nicolet iS 5 spectrometer (Thermo Fisher, United States). The samples were homogenized with dry KBr at a 1:100 ratio and compressed into pellets for measurement. Infrared spectra were recorded at a resolution of 4 cm^−1^ over the wavenumber range of 500–4,000 cm^−1^, with each spectrum representing an average of 32 scans.

### 2.10 Short-chain fatty acid levels

Short-chain fatty acids (SCFA) levels were determined using the previously established method (19). Briefly, 200 μL of fermentation fluid was subjected to liquid-liquid extraction using a 1:1 (v/v) mixture of 15% phosphoric acid and 1.6 mL of absolute ether. Analysis was performed on a SHIMADZU GC-MS system fitted with an Agilent DB-FFAP column (30 m × 0.250 mm × 0.25 μm). The carrier gas was helium at 1.0 mL/min (split ratio 10:1). The injector and ion source were set at 250 and 230 °C, respectively. For chromatographic separation, the column temperature started at 90 °C, was raised to 120 °C at 10 °C/min, then to 150 °C at 5 °C/min, and finally to 245 °C at 25 °C/min, holding for 2 min.

### 2.11 16 S rRNA gene and bioinformatics analysis

Majorbio Bio-Pharm Technology Co., Ltd. (Shanghai, China). sequenced the 16S rRNA gene of fermented fecal bacteria. The V3-V4 variable region of the bacterial 16S rRNA gene was amplified using specific primers 806R and 338F. Microbial community diversity was analyzed using methods such as the Chao index, Shannon index, and PCoA. The composition of the gut microbiota was evaluated at the phylum and genus levels.

### 2.12 Statistical analysis

The study was conducted with three independent replicates, with data reported as means. Data visualization and plotting were performed with Origin Pro 2021. Statistical analysis using DPS 7.05 software was applied to identify significant differences at a 95% confidence level (*p* < 0.05). Notably, the data for the unfermented samples (LRS3) were directly cited from the authors’ previously published studies (20) and used as baseline values in this study. The use of existing baseline data was intended to avoid duplicating experiments, allowing this study to focus on its original contribution—namely, the dynamic evolution of structure during the fermentation process.

## 3 Results and discussion

### 3.1 Fermentation characteristics

Changes in pH and OD_600_ during fermentation reflect alterations in the fermentation environment and microbial growth (21). All three samples showed a significant decrease in pH within 6 h, accompanied by a rapid increase in OD_600_, indicating continuous proliferation of normal/allergic fecal microbiota. Subsequently, as fermentation time extended, both pH and OD_600_ stabilized by 24 h (Supplementary Figure 1). Therefore, we selected the 6 and 24-h time points to analyze changes in starch structure, microbiota, and SCFA metabolites. This was consistent with the findings of Sun et al. (22) who observed a significant increase in the optical density (OD) of the fermentation broth at 6 h during the *in vitro* fermentation of butyrylated and recrystallized high-amylose corn starch. The growth rate slowed and stabilized by 24 h. This demonstrated that the enhanced carbohydrate metabolism was associated with an increase in bacterial biomass and more pronounced acidification of the fermentation medium.

Table 1 shows the changes in sugar derivatives produced by microbial utilization and degradation of LRS3 after fermentation. At 6 h of fermentation, glucose, maltose, maltotriose, and maltotetraose were detected in the fermentation broth. After continuing fermentation for 24-h, the contents of glucose, maltose, and maltotetraose decreased significantly, while maltotriose increased significantly. This indicated that glucose and maltose, being readily available carbon sources, were rapidly consumed through direct utilization by the microbial community (17). Maltotriose and maltotetraose are known intermediates produced during microbial starch utilization. As fermentation progressed, these intermediates were further degraded by the microbial community into oligosaccharides with lower polymerization degrees (23). The increase in maltotriose after 24 h of fermentation was due to the microbial utilization of starch and the subsequent hydrolysis of high-molecular-weight maltotetraose. Consequently, the concentration of maltotetraose decreased while the concentration of maltotriose increased.

**TABLE 1:** Concentration of sugar derivatives hydrolyzed by LRS3 during fermentation.

Sugar derivatives (mg/L)	LRS3-6	LRS3-24
Glucose	10.52 ± 0.11^a,C^	6.97 ± 0.10^b,C^
Maltose	60.32 ± 0.24^a,A^	57.20 ± 0.22^b,A^
Maltotriose	12.47 ± 0.14^b,B^	14.43 ± 0.13^a,B^
Maltotetraose	0.49 ± 0.04^a,D^	0.42 ± 0.01^b,D^
Maltopentose	ND	ND
Maltohexaose	ND	ND
Maltoheptaose	ND	ND

Different lowercase letters in the same row indicate significant differences in the sugar derivatives of different samples, *p* < 0.05. Different capital letters in the same column indicate significant differences in the sugar derivatives of the same sample, *p* < 0.05. ND, not detected.

### 3.2 Morphological properties

**FIGURE 1:**
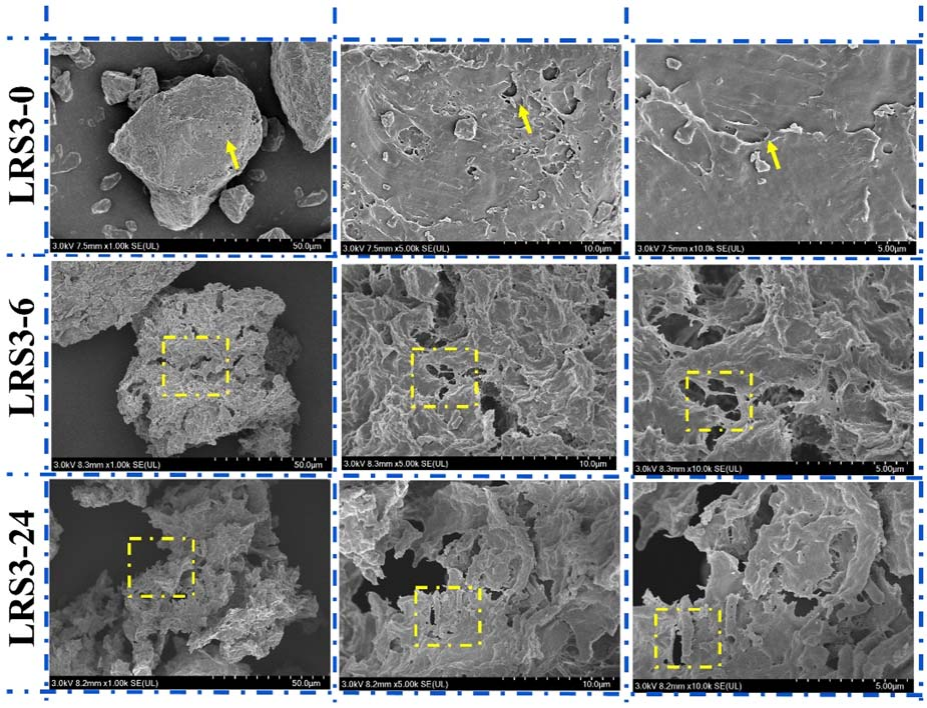
Scanning electron microscopy of LRS3 under *in vitro* simulated fermentation.

Figure 1 shows the morphological characteristics of LRS3 before and after fermentation. Before fermentation, LRS3 exhibited a scattered, lumpy, and irregular structure with fractured granules and a rough, uneven surface (see the yellow arrow), consistent with the findings of Zeng et al. (14). After fermentation, microbial communities eroded the starch granules. The starch granules in LRS3-6 and LRS3-24 were smaller, with a rougher surface exhibiting pores and cracks, presenting a hollowed-out appearance (see the yellow box). With longer fermentation duration, more severe morphological damage was observed, manifesting as increased fissures and smaller granular fragments. These morphological changes to the starch granules indicated that microbial degradation initiated at the granule surface. Research showed that microorganisms tended to attach to surfaces and form pores or cracks, which became the initiation points for diffuse disintegration (24).

### 3.3 Amylose content

**FIGURE 2:**
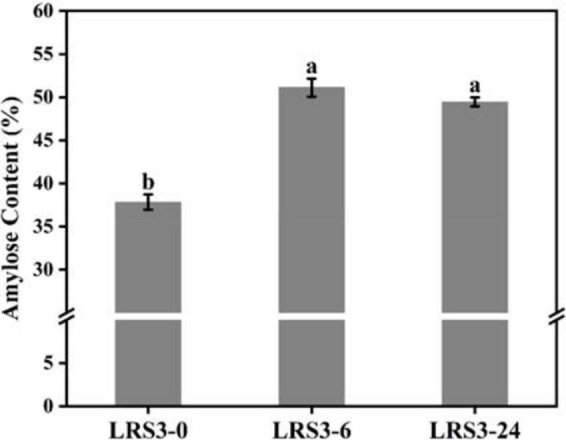
Amylose content of LRS3 under *in vitro* simulated fermentation.

The change in LRS3’s amylose content before and after *in vitro* fermentation is shown in Figure 2. After 6 h of fermentation, the amylose content of LRS3 significantly increased, while it slightly decreased after 24 h of fermentation. This indicated that the branched starch in LRS3 was more readily recognized and degraded by microbial enzyme systems during the early fermentation stage due to its complex branching structure. This resulted in the breakage of its short side chains and debranching, releasing more amylose fragments and causing the total amylose content to increase initially. In contrast, the amylose polymers in LRS3 were more resistant to enzymatic degradation due to stronger intramolecular hydrogen bonds and smaller specific surface area. This phenomenon was consistent with the findings of Lu et al. (25) who suggested that the preferential degradation of amylopectin by microbial extracellular enzymes leads to an increase in amylose content with lower average degree of polymerization, thereby altering the ratio between the two starch components. Bian et al. (26) also reported increased amylose content within specific fermentation time windows. As fermentation progressed to 24 h, the accumulation of SCFAs and the resulting pH decrease may have partially inhibited microbial enzyme activity, slowing the degradation of amylose. Consequently, its content slightly decreased showed no significant change. This result also was in agreement with the findings of Ye et al. (27) who observed that the amylose content in sweet potato starch decreased when the pH of the fermentation system was continuously lowered to a certain level.

### 3.4 Chain length distribution of amylopectin

**FIGURE 3:**
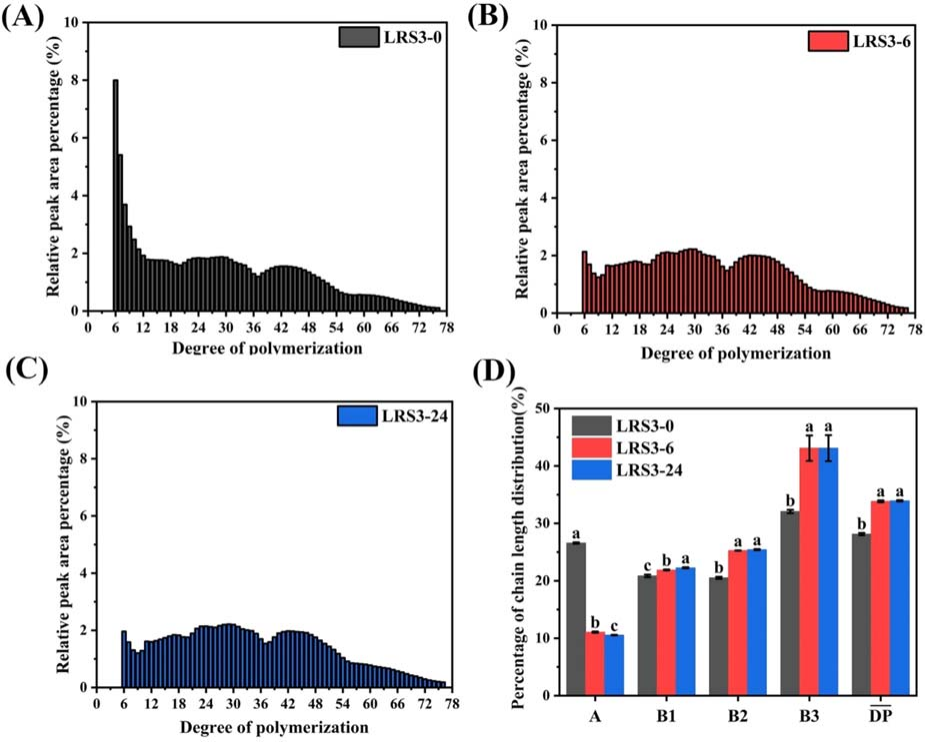
Chain length distribution of amylopectin of LRS3 under in vitro simulated fermentation. Different lowercase letters indicate significant differences in the proportion of each chain length distribution at different fermentation times for the same sample, *p* < 0.05. **(A)** LRS3-0. **(B)** LRS3-6. **(C)** LRS3-24. **(D)** Proportion of classified amylopectin chains.

The chain length distribution of LRS3 amylopectin before and after fermentation was analyzed by ion chromatography. Amylopectin chains were categorized into four groups based on degree of polymerization (DP): A-chains (DP 6–12), B1-chains (DP 13–24), B2-chains (DP 25–36), and B3-chains (DP > 36) (28). As shown in Figure 3, during fermentation, the content of short A-chains in LRS3 decreased significantly, while the proportion of longer B-chains and the average chain length showed an increasing trend. This can be attributed to the fact that the outer A-chains, due to their shorter length and higher accessibility, are more readily degraded and utilized by microbial enzymes. These results suggested that the cleavage of short side chains from amylopectin released shorter linear chains, which was consistent with the observed increase in amylose content at 6 h of fermentation, as mentioned earlier. This finding aligns with the report by Lv et al. (29), who also observed that an increase in amylose content in corn starch samples with varying amylose contents corresponded to a reduction in A-chains and an increase in B2- and B3-chains within the starch. However, when fermentation was extended to 24 h, the lack of a significant further increase in B2- and B3-chains could be explained by the suppressed microbial enzyme activity under the low-pH conditions in the fermentation broth. This was also consistent with the previously discussed trends in amylose content.

### 3.5 Crystalline structure analysis

**FIGURE 4:**
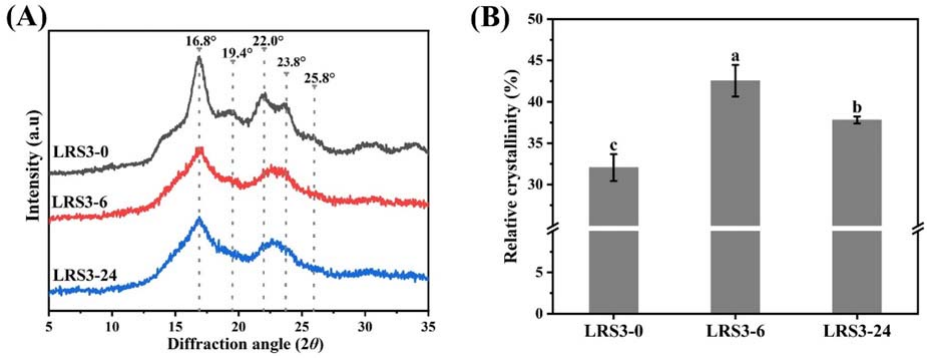
**(A)** X-ray diffraction patterns of LRS3 under *in vitro* simulated fermentation. **(B)** Relative crystallinity of LRS3 under *in vitro* simulated fermentation.

Amylose and amylopectin form structurally stable crystalline regions (ordered layered stacking) and loosely amorphous regions (disordered random distribution) based on their spatial arrangement. The X-ray diffraction patterns of LRS3 before and after *in vitro* fermentation, as shown in Figure 4A. The primary diffraction peaks for LRS3-0, LRS3-6, and LRS3-24 were 16.8°, 19.4°, 22.0°, and 23.8°, respectively, with a weak peak at 25.8°, indicating a typical B-type crystalline structure. This indicated that fermentation did not alter the crystal type of LRS3, which is consistent with the findings of Liu et al. (24).

The dynamic changes in relative crystallinity of LRS3 during *in vitro* fermentation, as shown in Figure 4B. After fermentation, the relative crystallinity of LRS3 exhibited an initial significant increase followed by a significant decrease. This change can be attributed to the preferential degradation of starch structure by microorganisms during fermentation: In the early fermentation stage (6 h), the microbial community prioritized utilizing structural components in the amorphous region, such as the branch points of amylopectin, short amylopectin chains, and disordered amylose fragments. The depletion of this region led to a relative increase in the proportion of crystalline regions, thereby causing a significant rise in relative crystallinity. As fermentation progressed (6–24 h), microbial activity extended from the amorphous regions into crystalline zones, disrupting intermolecular hydrogen bonds that maintain crystal structure and interfering with the ordered arrangement of starch chains. This ultimately led to the disintegration of crystalline structures, resulting in a significant decrease in relative crystallinity. This was consistent with the findings of Zhao et al. (30), who found that the crystallinity of lentil resistant starch did not increase continuously with fermentation time during *in vitro* faucal fermentation, but showed a slight decrease after 24 h. Notably, the crystallinity of LRS3-6 and LRS3-24 was significantly higher than that of LRS3-0. Longer amylopectin chains stabilized the crystalline layered structure, and starches with longer amylopectin chains tended to exhibit higher crystallinity than those with shorter chains. This was because longer chains could form more extensive hydrogen bonds and interact more strongly with adjacent chains, consistent with the earlier findings on amylopectin chain length distribution.

### 3.6 FTIR spectroscopic analysis

**FIGURE 5:**
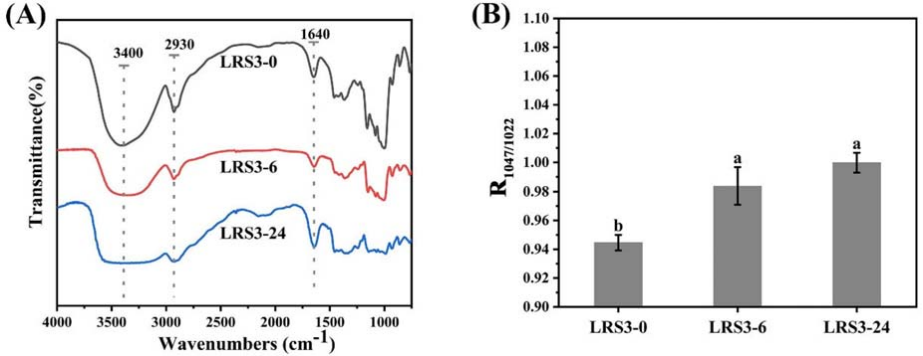
**(A)** FTIR spectra of LRS3 under *in vitro* simulated fermentation. **(B)** 1,047/1,022 ratio at the FTIR vibration of LRS3 under *in vitro* simulated fermentation.

Figure 5A shows the FTIR spectrum of LRS3 before and after fermentation. By observing the hydroxyl absorption band in the 3,800–3,000 cm^−1^ range, it was found that during fermentation, the absorption peak in this band broadened. This indicated that fermentation reduced the density of hydroxyl groups within the LRS3 starch molecules, consumed hydrogen bonds, weakened intermolecular hydrogen bonding forces and enhancing the accessibility of starch to microbial communities and their enzymes. This finding was consistent with the results of the previously reported SEM analysis.

Studies have demonstrated that the ratio of infrared absorption peaks at 1,047 cm^−1^ and 1,022 cm^−1^ (R1047/1022) can be used to characterize the short-range order in starch systems (31). Figure 5B reflects the dynamic changes in the short-range order of LRS3 before and after fermentation. The short-range order of LRS3 consistently increased after fermentation. This may be attributed to the degradation of non-compact amorphous regions primarily by enzymes produced by gut microbiota. The resulting shorter-chain starch molecules subsequently aggregated to form ordered microcrystals, thereby enhancing the short-range order of the molecules. This observation was supported by the findings reported by Zhao et al. (30). They also observed this pattern in their study of multiscale structural changes in mung bean resistant starch during *in vitro* dynamic fermentation. Interestingly, different from the long-range order (relative crystallinity), the short-range order (LRS) of LRS3 increased continuously, albeit not significantly, during fermentation from 6 to 24 h. This was due to the persistence of local double-helix structures and the rearrangement of short-chain fragments generated during fermentation into new locally ordered structures. However, this was insufficient to reverse the decreasing trend in LRS3’s long-range order. It was shown that the presence of short-range order does not necessarily imply the existence of long-range order. Short-range ordered structures may determine the hydrolytic properties of starches containing amorphous or low-order structures, but they may not be significantly correlated with the hydrolytic properties of starches possessing relatively ordered long-range structures (32).

### 3.7 Effect of LRS3 fermentation on SCFAs

**FIGURE 6:**
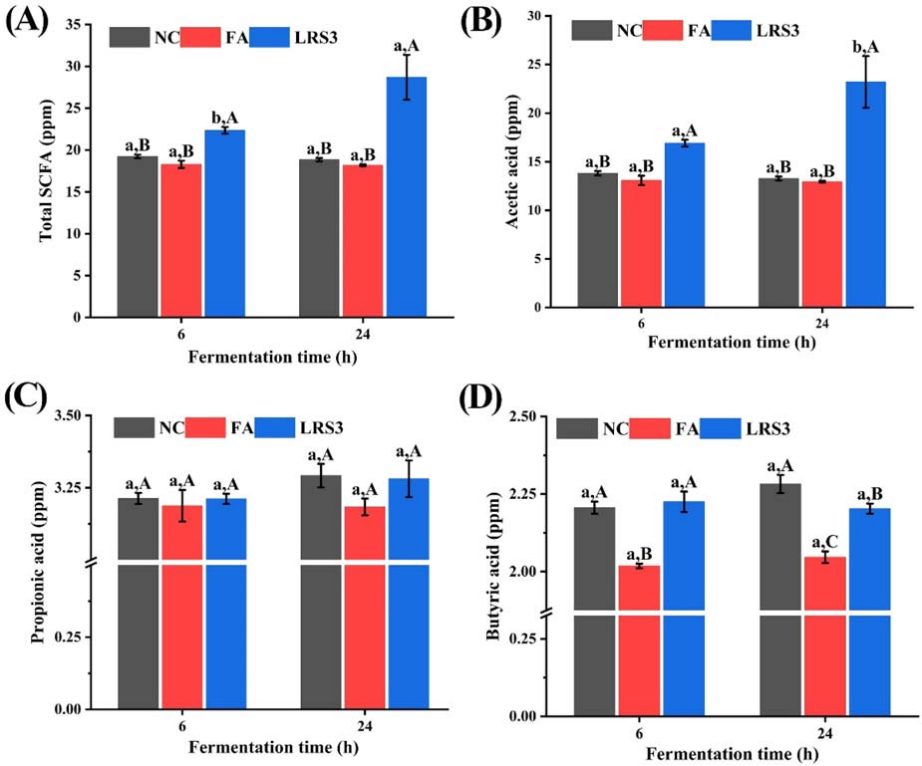
**(A)** Total SCFA levels during *in vitro* fermentation in each group. **(B)** Acetic acid levels during *in vitro* fermentation in each group. **(C)** Propionic acid levels during *in vitro* fermentation in each group. **(D)** Butyrate levels during *in vitro* fermentation in each group. Different lowercase letters for the same sample at different fermentation time points indicate significant differences (*p* < 0.05); different uppercase letters at the same fermentation time point between samples indicate significant differences (*p* < 0.05).

The gut microbiota can break down and convert indigestible carbohydrates within the host into absorbable monosaccharides or SCFAs, providing energy for colonic cells and promoting the development of intestinal epithelial cells. The changes in acetate, propionate, and butyrate production among different treatment groups (NC, FA, and LRS3) during *in vitro* simulation of normal and allergic mouse fecal fermentation are shown in Figure 6. The figure showed that compared to the LRS3 group, the NC and FA groups exhibited lower total short-chain fatty acid production, indicating that LRS3, as a carbohydrate substrate, could be efficiently fermented and utilized by gut microbiota. Previous study found that flatbreads subjected to type II sourdough fermentation—a process known to restructure cereal carbohydrates—significantly improved gut microbiota composition and boosted short-chain fatty acid production during subsequent *in vitro* fecal fermentation. Such findings reinforce the concept that controlled microbial degradation of complex carbohydrate matrices, whether in cereal-based foods or isolated resistant starch, consistently favors the proliferation of acetate-producing commensals (33–35). Notably, the trend of acetate changes over fermentation time highly correlated with total SCFA content across all groups. Furthermore, the pattern observed for total SCFAs was consistently mirrored by significant differences in acetate levels between treatment groups at any fermentation time point, indicating that acetate serves as the predominant metabolite throughout fermentation and that its production and metabolism play a dominant role. Stepwise analysis revealed that, as fermentation time increased, acetate levels rose significantly more in the LRS3 group than in the NC and FA groups. In contrast to the acetate pattern, butyrate levels in the LRS3 group increased more slowly than in the FA group. At 6 h and 24 h, the LRS3 group had significantly higher butyrate levels than the FA group. This suggested that LRS3, when used as a fermentation substrate, not only promoted the production of acetate by the gut microbiota in allergic mice, but also enhanced the synthesis of butyrate.

Based on these findings, it is speculated that LRS3 may promote substantial acetate production, with a portion converted to butyrate via the butyryl-CoA:acetyl-CoA transferase pathway during early fermentation (36). As fermentation progresses, the decrease in fermentation broth pH may partially suppressed acetate-CoA transferase activity, thereby limiting acetate-to-butyrate conversion in the later stages. Therefore, in the fermentation system regulated by LRS3, the increase in acetate represents the most critical change, governing the metabolic flux and accumulation patterns of all short-chain fatty acids.

### 3.8 Effect of LRS3 fermentation on the composition of gut microbiota

**FIGURE 7:**
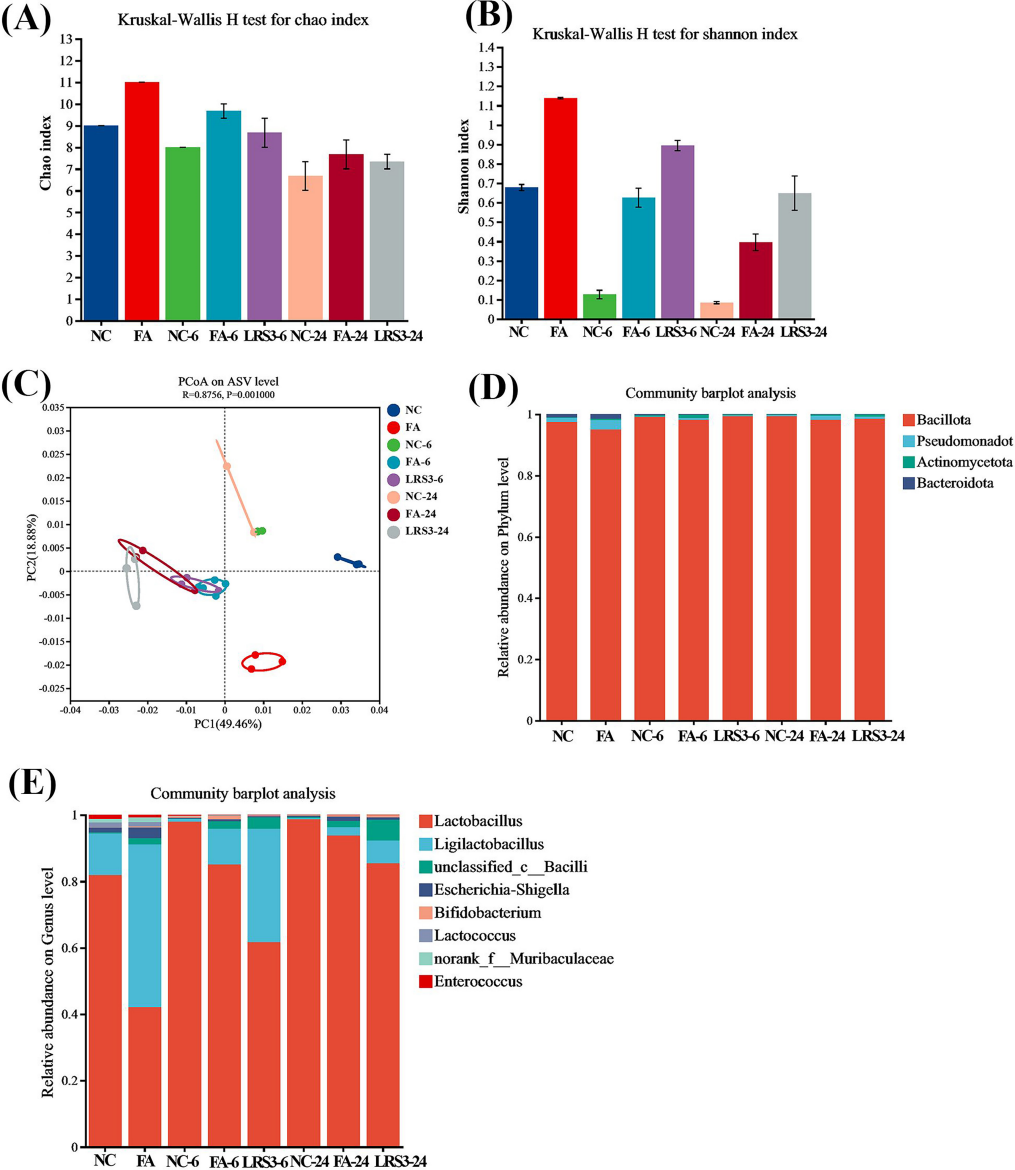
Effects of LRS3 on the overall structural composition of fecal microbiota in food-allergic rats. **(A)** Shannon index. **(B)** Simpson index. **(C)** PCoA analysis. **(D)** Phylum-level classification analysis of bacterial samples. **(E)** Genus-level classification analysis of bacterial samples.

The overall differences in microbial community structure at the genus level, as analyzed based on 16S rRNA gene sequences, are shown in Figure 7. Alpha diversity analysis was employed to evaluate species richness and diversity within microbial communities: the Chao index primarily reflects community richness, while the Shannon index focuses more on community diversity. Both richness and diversity decreased with extended fermentation time across all experimental groups (Figures 7a,b). Notably, at fermentation times of 6 h and 24 h, the addition of LRS3 did not significantly affect the alpha diversity of the microbial community in the FA group compared to the FA group. These results suggested that the fermentation process of LRS3 might favor the enrichment of specific bacteria rather than enhancing the overall diversity of the microbial community. This finding was consistent with the previous study (37), which found that fermentation of Atractylodes chinensis (DC.) Koidz. polysaccharides in an *in vitro* simulated digestion-fermentation model also did not significantly alter the gut microbiota diversity of type 2 diabetic patients, but specifically enriched Bifidobacterium.

Beta diversity analysis was used to assess the structural similarity of the composition of the gut microbiota among the different rat groups (see Figure 7C). Regardless of fermentation duration, principal coordinate analysis (PCoA) plots revealed that the NC group was distributed in the second quadrant, while the FA group primarily occupied the third and fourth quadrants. This indicated significant differences in microbial community structure between the NC and FA groups. At 6 -h fermentation, the PCoA regions of the LRS3 group and FA group partially overlapped, suggesting that LRS6 supplementation had a minor effect on the overall microbial composition of allergic rats during the early fermentation phase. However, at 24 -h fermentation, the LRS3 group and FA group formed two distinct clusters, indicating that prolonged fermentation gradually induced significant changes in the microbial community structure of allergic rats by LRS3.

**FIGURE 8:**
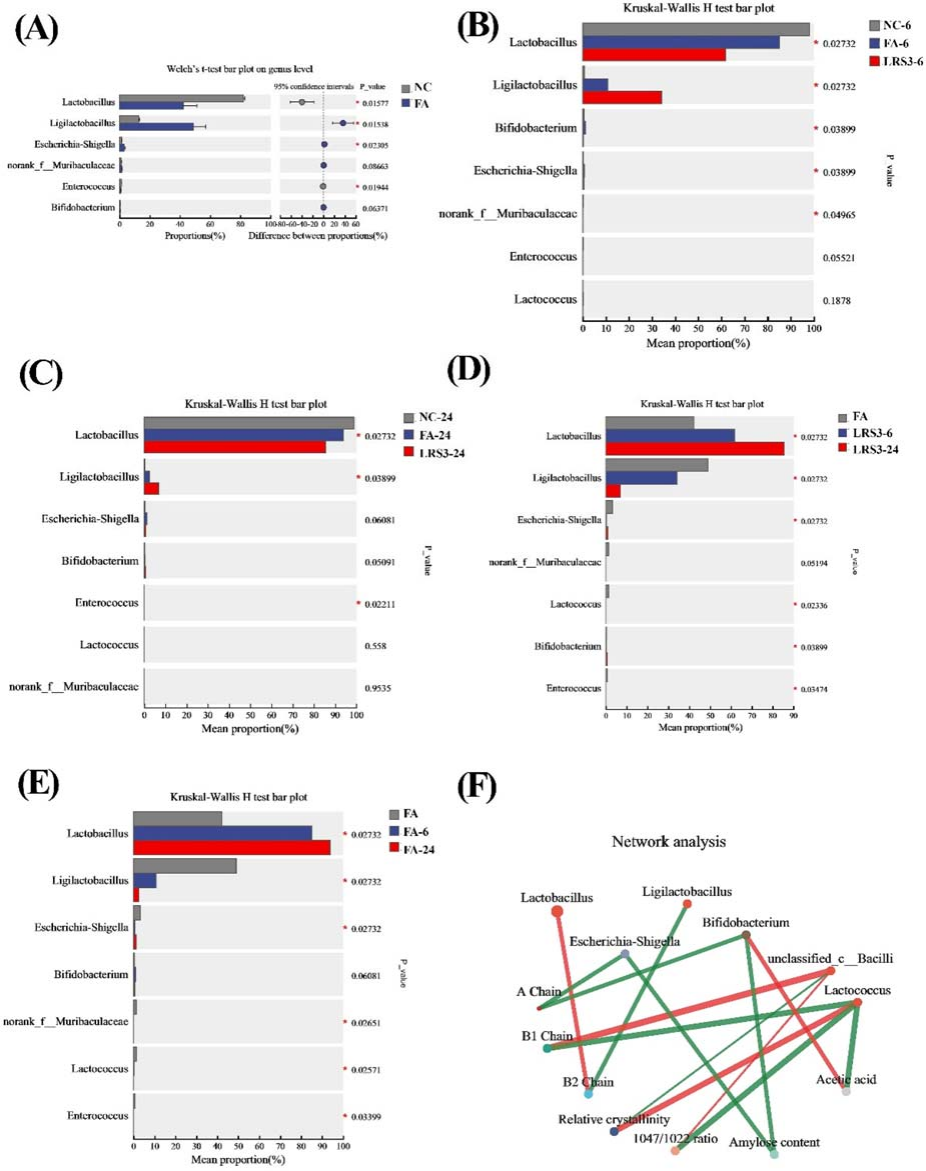
**(A–E)** Genus-level differential microbiome analysis among groups. **(F)** Structure-microbiome-SCFAs correlation network diagram for LRS3 group in *in vitro* fermentation.

Microbial community composition at the phylum and genus levels are shown in Figures 7D,E. At the phylum level, microbial communities across all groups were primarily composed of the phyla Bacillota, Pseudomonadota, Actinomycetota, and Bacteroidota. At the genus level, major genera included Lactobacillus, Ligilactobacillus, unclassified_c_Bacilli, Escherichia-Shigella, Bifidobacterium, Lactococcus, norank_f_Muribaculaceae, and Enterococcus. Further comparison between the NC group and the FA group (pre-fermentation) revealed differentially abundant genera including Lactobacillus, Ligilactobacillus, Escherichia-Shigella, and Enterococcus, with significant increases in Ligilactobacillus and Escherichia-Shigella observed in allergic stool samples (Figure 8A). Research indicated that shigellosis is an invasive acute lower intestinal disease affecting millions globally each year and causing approximately one million deaths (38). Interestingly, at 6 -h fermentation, the relative abundance of Escherichia-Shigella in the FA group remained significantly higher than in the NC group, but its relative abundance decreased with the introduction of LRS3, a trend also observed at 24 -h fermentation (Figures 8B,C). Furthermore, as fermentation progressed, the relative abundance trend of the differentially abundant genus Bifidobacterium in the LRS3 group was opposite to that in the FA group, particularly at 24 h where Bifidobacterium significantly increased in the LRS group (Figures 8D,E; Supplementary Figure 3). Further analysis of the relative abundance ratio of Bifidobacterium/Escherichia-Shigella across groups (Supplementary Figure 4) revealed that compared to the unfermented state, this ratio significantly increased in both the LRS3-6 h and LRS3-24 h groups. Moreover, at 24 h of fermentation, the ratio in the LRS3 group was significantly higher than that in the FA group. Based on these findings, we hypothesized that LRS3 may exert its anti-allergic effects by promoting Bifidobacterium proliferation and suppressing Escherichia-Shigella. Research indicates that Bifidobacterium, belonging to the Actinobacteria phylum, is considered a key beneficial gut microorganism due to its ability to hydrolyze carbohydrates and produce SCFA.

Additionally, Spearman’s correlation coefficient was used to analyze associations between dominant microbial communities and SCFAs in LRS3 group. Supplementary Figure 5 showed that Bifidobacterium, Lactobacillus, and Escherichia-Shigella were positively correlated with acetate; Lactobacillus was positively correlated with propionate; and Ligilactobacillus was positively correlated with butyrate. Statistically significant positive correlations were found only between Bifidobacterium and acetate. Integrating these findings with previous microbiome analyses, LRS may alleviate allergies by promoting Bifidobacterium proliferation. We therefore hypothesize that Bifidobacterium may exert anti-allergic effects by utilizing LRS3 to produce acetate. Previous studies indicate that Bifidobacterium primarily or exclusively produces acetate during fermentation (39). Acetic acid modulated inflammasomes via GPR43 and GPR109A, producing IL-18 to promote intestinal barrier repair., thereby further inhibiting inflammatory responses and allergic disease development (40).

To further explore the relationship among structural characteristics, microbial communities, and SCFAs in the LRS3 group during *in vitro* fermentation, we constructed a network diagram of the three factors using Spearman’s correlation (Figure 8F). In the LRS3 group, amylose content showed a negative correlation with Escherichia-Shigella and Bifidobacterium. The 1,047/1,022 ratio showed a negative correlation with Lactococcus. Relative crystallinity exhibited a positive correlation with Lactococcus. The A-chain of branched starch showed a negative correlation with Escherichia-Shigella and Bifidobacterium. The B1-chain exhibited a negative correlation with Lactococcus. The B2-chain showed a negative correlation with Ligilactobacillus and a positive correlation with Lactobacillus. These results indicate that the fine structural characteristics of LRS3 constitute the material basis for driving microbial community restructuring and exerting its allergy-alleviating function. Among these, both amylose and amylopectin A chain showed negative correlations with Escherichia-Shigella and Bifidobacterium, suggesting they may be competitively acquired by beneficial bacteria, thereby limiting the growth of pathogenic bacteria in their ecological niche. Meanwhile, more complex amylose B chains (e.g., B1, B2) exhibited distinct correlations with genera such as Lactococcus and Ligilactobacillus, reflecting functional differentiation among microbes in utilizing complex carbohydrates. Crucially, this study successfully linked structure-driven microbial community changes to metabolic phenotypes (SCFAs). Among all major bacterial groups, only Bifidobacterium showed a significant positive correlation with acetate content. This finding further confirms that LRS3 selectively promotes Bifidobacterium as the dominant functional microorganism through its structural properties. Subsequently, via acetate metabolism, it governs the metabolic flux and microbial community structure within the fermentation system, ultimately exerting its anti-allergic effects.

### 3.9 Effect of LRS3 fermentation on microbial function prediction

**FIGURE 9:**
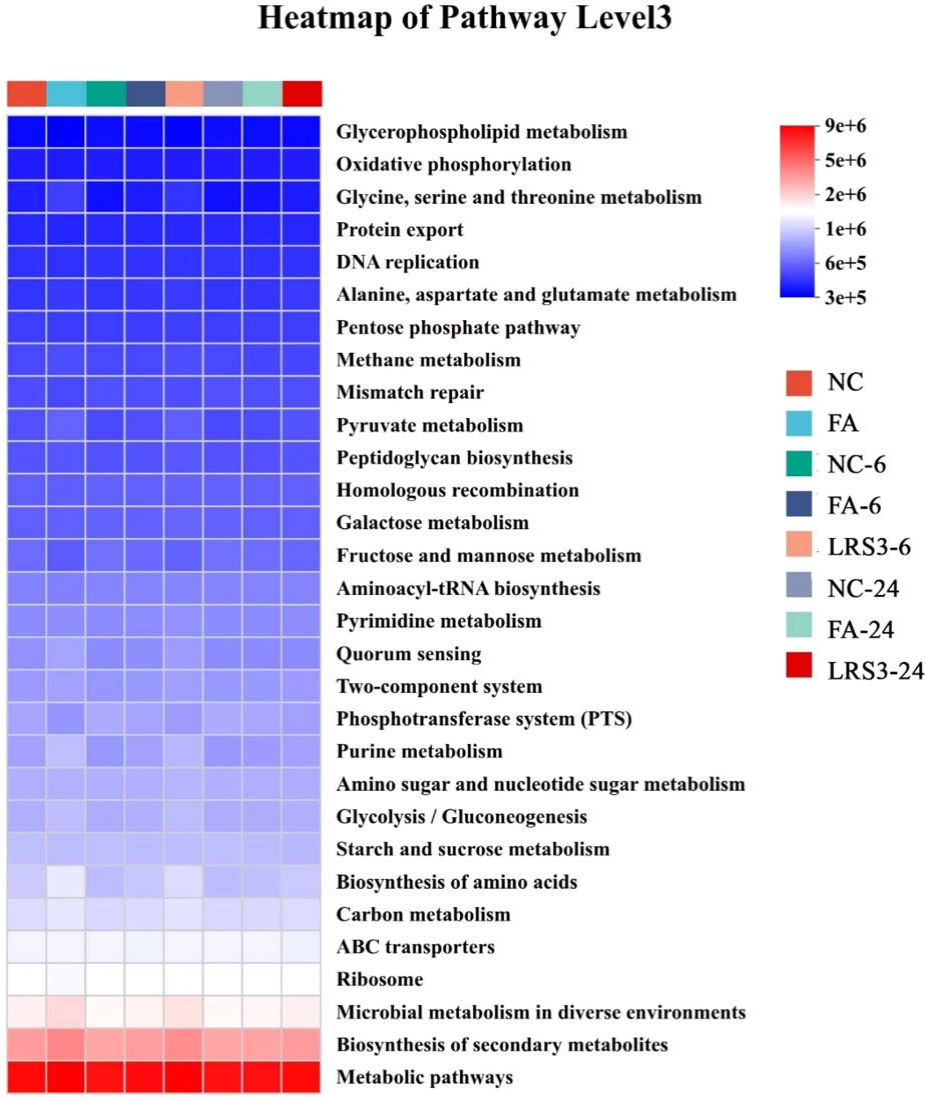
PICRUSt-predicted KEGG function abundance.

Based on the KEGG database, PICRUSt2 prediction of the microbial metabolic pathways in different fermentation systems was performed, as shown in Figure 9. The core metabolic pathways of all samples displayed a distinct hierarchical distribution pattern. Among them, Metabolic pathways, Biosynthesis of secondary metabolites, and Microbial metabolism in diverse environments, as the highest-level core pathways, exhibited prominent red high-abundance signals in the LRS3 group, and their intensities were consistently higher than those in the allergic group at the same time points, indicating that LRS3 intervention significantly activated the overall metabolic activity of the microbiota. Among the pathways directly related to carbohydrate utilization, Starch and sucrose metabolism, Carbon metabolism, Glycolysis/Gluconeogenesis, and Pyruvate metabolism all exhibited higher abundances in the LRS3 group. Specifically, the specific activation of Starch and sucrose metabolism served as a key functional module for beneficial bacteria such as Bifidobacterium to degrade resistant starch. LRS3, as a type 3 lotus seed resistant starch, could be hydrolyzed into oligosaccharides and glucose by hydrolytic enzymes secreted by beneficial bacteria such as Bifidobacterium. The synergistic upregulation of Carbon metabolism and Pyruvate metabolism occurred because the monosaccharides produced from resistant starch degradation entered the carbon metabolism and glycolysis pathways to generate pyruvate. Pyruvate was a key precursor for the production of short-chain fatty acids, especially acetate. The sustained high abundance of these two pathways in the LRS3 group indicated that the carbon metabolic flux of the microbiota was efficiently directed toward pyruvate production, providing sufficient substrates for subsequent acetate synthesis. This corresponded directly to the previous result that “the acetate content in the LRS3 group increased continuously and significantly.” The above results indicated that LRS3 intervention could regulate the carbon metabolic flux and promote acetate production by targeting the activation of metabolic pathways related to resistant starch degradation, thereby exerting a regulatory effect on the gut microecology.

## 4 Conclusion

This study provided evidence of a distinct structure-function relationship in the regulation of *in vitro* fecal fermentation by LRS3 in food-allergic rats. LRS3 underwent significant structural disintegration, including particle erosion, initial amylose degradation, and eventual breakdown of crystalline regions. These physical and molecular alterations generated a fermentable substrate selectively utilized by the gut microbiota. Although LRS3 fermentation did not enhance overall microbial diversity, it triggered a directed reshaping of community structure. The most striking outcome was the marked enrichment of Bifidobacterium alongside the suppression of Escherichia coli-Shigella. A significant positive correlation was observed between Bifidobacterium abundance and acetate (the predominant SCFAs), indicating this species as a core mediator in LRS3-induced allergy mitigation. We proposed that during fermentation, the structure of LRS3 underwent changes, thereby becoming the preferred nutrient source for Bifidobacterium. We will subsequently validate, through in vivo animal experiments, that the proliferation of Bifidobacterium and the acetate they produce are the key drivers of the protective effect against food allergies. This study highlights the potential of utilizing structurally defined resistant starch as a precision prebiotic to manage food allergies through targeted regulation of the microbiome.

## Data Availability

The original contributions presented in this study are included in the article/Supplementary material, further inquiries can be directed to the corresponding authors.
