# Supplementary material for: Structural evolution of lotus seed resistant starch during in vitro fecal fermentation in food-allergic rats modulates gut microbiota and SCFAs
Source: Front Nutr. 2026 Jun 30;13:1870906. doi: 10.3389/fnut.2026.1870906 (PMC13367886; doi:10.3389/fnut.2026.1870906)

**Supplementary Figure captions**

Figure S1 The changes in pH values of fermentation broths during in vitro simulated fermentation processes

Figure S2 The changes in OD_600_ values of fermentation broths during in vitro simulated fermentation processes

Figure S3 Relative abundance of *Bifidobacterium* between groups. Different lowercase letters for the same sample at different fermentation time points indicate significant differences (*p* < 0.05); different uppercase letters at the same fermentation time point between samples indicate significant differences (*p* < 0.05).

Figure S4 Relative abundance ratios of *Bifidobacterium/Escherichia coli-Shigella* between groups. Different lowercase letters for the same sample at different fermentation time points indicate significant differences (*p* < 0.05); different uppercase letters at the same fermentation time point between samples indicate significant differences (*p* < 0.05).

Figure S5 Spearman correlation analysis between SCFAs levels and gut microbiota genus-level composition during in vitro fermentation. **p* < 0.05.

Figure S1





Figure S2





Figure S3





Figure S4





Figure S5


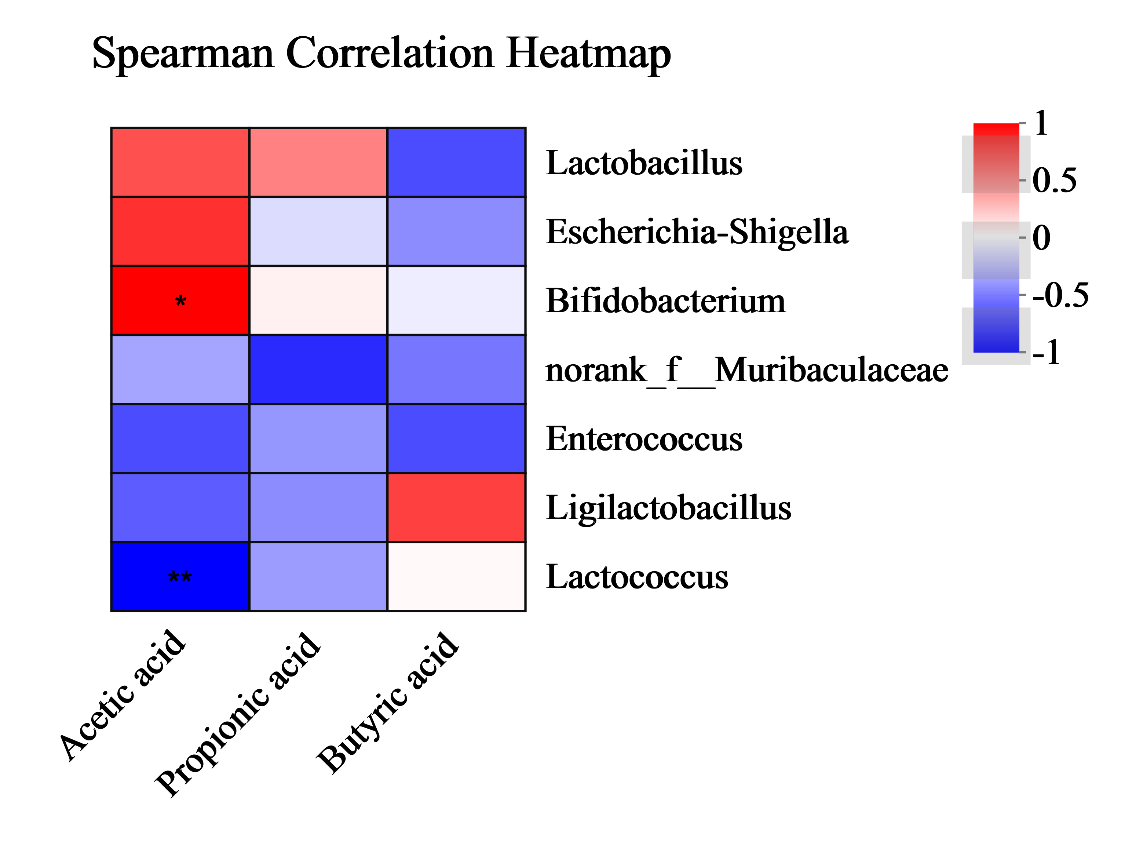

Supplement: Supplementary file 1 [file Supplementary_file_1.docx]
